# Developmental Status of Five-Year-old Children with Very-Low-Birth-Weight 

**DOI:** 10.22037/ijcn.v15i2.18702

**Published:** 2021

**Authors:** Reza SHARAFI, Afagh HASSANZADEH RAD, Narges AKRAMI, Vahid AMINZADEH

**Affiliations:** 1 1.Pediatric Diseases Research Center, Guilan University of Medical Sciences, Rasht, Iran.

**Keywords:** Developmental delay, Child, ASQ, VLBW

## Abstract

**Objective:**

Some previous studies have reported the improved survival of very-low-birth-weight (VLBW) neonates with no disabilities. However, 16% of these neonates have developmental disorders. Considering the lack of research on the developmental status of five-year-old VLBW children and the importance of early detection and treatment, in this study, we aimed to assess the developmental status of five-year-old VLBW children.

**Materials & Methods:**

This historical cohort study was conducted on five-year-old children. The participants were divided into VLBW and normal-birth-weight (NBW) groups. Data were gathered using the Ages and Stages Questionnaire (ASQ). This questionnaire consisted of five developmental domains, including communication, gross motor, fine motor, problem-solving, and personal/social skills. Data were reported by measuring descriptive statistics, including mean, standard deviation, number, and percentage, and analyzed by Mann-Whitney U test and independent t-test in SPSS version 22.

**Results:**

A total of 106 five-year-old children, including two groups of VLBW and NBW, participated in this study. The results of Mann-Whitney U test showed a significant difference between the groups regarding the scores of communication (P=0.002), gross motor (P<0.001), fine motor (P<0.001), and problem-solving (P<0.001) skills. However, no significant difference was found between the groups regarding the personal/social developmental status (P=0.559).

**Conclusion:**

According to the results, a higher risk of developmental delay was observed in VLBW infants as compared to NBW neonates; therefore, it is recommended to perform developmental screening tests for timely detection of high-risk children and early diagnostic and therapeutic interventions.

## Introduction

Very low birth weight (VLBW) is defined as the first neonatal birth weight less than 1500 g. Although today, the mortality rate of VLBW neonates has decreased significantly following the development of intensive care medicine, multiple complications still occur. Cerebral palsy is the most common neurological complication of VLBW neonates (1). Besides, cognitive disorder, blindness, deafness, memory dysfunction, strabismus, speech delay, dyslexia, and behavioral disorders can be mentioned as other significant complications (2).

There are major concerns about the increased survival rate of VLBW neonates, which can increase the rate of neurological disabilities (3). Although some previous studies have reported the improved survival of these neonates with no disabilities (4), 16% of them show developmental disorders (5). In other words, behavioral and developmental disorders are the most common problems during childhood. However, if diagnostic and therapeutic interventions are applied in preschool years, many problems can be resolved. 

Speech delay is the most common measurable developmental disorder. Relatively 25% of children with developmental disorders are detected before school attendance; therefore, most of them are deprived of early interventions. The American Academy of Pediatrics (AAP) recommended screening and developmental surveillance for improvement and early detection of developmental problems in primary care units. Generally, developmental screening requires standard facilities with high sensitivity and specificity, and the accuracy of screening tests (sensitivity and specificity) is estimated at 70-80% (6).

Screening tests can be carried out in two ways. Some tests, such as Denver II or Bayley scale, must be administered by trained personnel, while other tests, such as Ages and Stages Questionnaire (ASQ), are administered by the parents. Undoubtedly, parental attendance in the evaluation process and use of their information are important for childhood development. According to previous investigations, the parents have the ability to assess their children’s function, and their concerns regarding the child’s developmental status are important (7). Besides, there is a significant relationship between the parents and trained personnel’s estimations of child development (8).

The ASQ is a test for assessing 4- to 60-month-old children, with 75% and 100% sensitivity and 95% and 90% specificity in high-risk and normal populations, respectively (9). The validity of this test is estimated at 76-88% (10). This questionnaire consists of five developmental domains, including communication, gross motor, fine motor, problem-solving, and personal/social skills. The selected questions were designed to show a developmental quotient of 75-100% (11).

Considering the lack of investigations on the developmental status of five-year-old VLBW children and the importance of early detection and treatment, in this study, we aimed to assess the developmental status of five-year-old VLBW children.

## Materials & Methods

This historical cohort study was conducted on five-year-old children. The participants were divided into VLBW (<1500 g) and NBW groups (>2500 g). Ethical approval was obtained from Guilan University of Medical sciences (IR.GUMS.REC.1394.470). Data were gathered by the ASQ, which was adjusted by the Vice Chancellor for Health of the Iranian Ministry of Health. This questionnaire consists of five developmental domains, including communication, gross motor, fine motor, problem-solving, and personal/social skills. The researchers designed the images and questions to be easily understandable, even for parents with a low educational level. Each question has three possible answers: (1) “Yes” (the child has the ability to completely do the skill); (2) “sometimes” (the child began to do the skill); and (3) “not yet” (the child does not do the skill). 

Cutoff points were used to assess the need for follow-up and further assessment. These cutoff points for Iranian children were previously reported by the Iranian Ministry of Health. They were determined regarding the function of each domain in the majority of same-aged children (10). The “yes”, “sometimes”, and “not yet” answers were assigned 10, 5, and 0 scores, respectively. If the score was ≥-1 standard deviation (SD) of the cutoff point, the child had no problems, while if it was ≤-2 SD of the cutoff point, he/she needed further assessment. A score between -1 SD and -2 SD of the cutoff point indicated the need for further practice of the skill; assessment was also essential two weeks later. If after two weeks, the score was not ≥-1 SD, further assessment was needed (suspected case).

Data were reported by measuring descriptive statistics, including mean, SD, number, and percentage, and analyzed by Mann-Whitney U test and independent t-test in SPSS version 22. The odds ratio and relative risk were also assessed in this study. P-value <0.05 was considered statistically significant, and a 95% confidence interval was used. 

## Results

In this study, 106 five-year-old children were examined, including two groups of VLBW children (birth weight <1500 g) and NBW children. Overall, 50 and 56 children were boys (47.2%) and girls, respectively, and there was no significant difference between the groups regarding sex (P=0.24). The mean birth weight of VLBW and NBW children was 1277.10±173.20 and 3136.70±358.50 g, respectively. The mean recent weight of VLBW and NBW children was also 15.80±2.86 and 20.40±4.99 kg, respectively; the results showed a significant difference between the groups. Also, the mean difference between the groups regarding the mean weight was 4.70±0.79 kg (P<0.001).

The developmental status of the groups showed higher rates of developmental delay in fine motor, problem-solving, gross motor, and communication skills of the VLBW children as compared to the NBW children (Table 1).

The results of Kolmogorov-Smirnov test did not indicate the normal distribution of the groups regarding the developmental domains (P<0.05), and non-parametric Mann-Whitney U test indicated a significant difference between the groups regarding the score of communication (P=0.002), gross motor (P<0.001), fine motor (P<0.001), and problem-solving (P<0.001) skills. However, no significant difference was found between the groups regarding the social/personal developmental status (P=0.559) (Table 2).

Besides, comparison of the scores of different developmental domains in the groups regarding sex showed a significant difference in all of the mentioned domains, except the social/personal developmental status (Table 3). 

**Table 1:** comparing developmental status in groups

**Developmental status**		**groups**				**P**
		**VLBW**		**NBW**		
**Communication**	**impaired**	**5**	**9.4%**	**0**	**0%**	**028/0**
	**normal**	**48**	**6/90%**	**53**	**100%**	
**Gross motors**	**impaired**	**6**	**3/11%**	**0**	**0%**	**013/0**
	**normal**	**47**	**7/88%**	**53**	**100%**	
**Fine motors**	**impaired**	**17**	**1/32%**	**2**	**8/3%**	**0001/0**
	**normal**	**36**	**9/67%**	**51**	**2/96%**	
**Socio-personal**	**impaired**	**4**	**5/7%**	**1**	**9/1%**	**181/0**
	**normal**	**49**	**5/92%**	**52**	**1/98%**	
**Problem-solving**	**impaired**	**10**	**9/18%**	**0**	**0%**	**001/0**
	**normal**	**43**	**1/81 %**	**53**	**100%**	

**TABLE 2:** comparing scores of developmental domains in groups

**Developmental status**	**groups**	**NUM**	**MEAN**	**SD**	**Mean Rank**	**P**
**Communication**	**VLBW**	**53**	**9057/52**	**76152/12**	**08/45**	**0020/0**
	**NBW**	**53**	**9245/57**	**31575/3**	**92/61**	
**Gross motors**	**VLBW**	**53**	**4528/51**	**95707/14**	**17/43**	**0001/0**
	**NBW**	**53**	**5849/58**	**44983/3**	**83/63**	
**Fine motors**	**VLBW**	**53**	**0189/40**	**83629/15**	**74/37**	**0001/0**
	**NBW**	**53**	**4340/55**	**88930/8**	**26/65**	
**Socio-personal**	**VLBW**	**53**	**8679/51**	**44939/13**	**85/51**	**5590/0**
	**NBW**	**53**	**9057/54**	**14133/5**	**15/55**	
**Problem-solving**	**VLBW**	**53**	**2358/40**	**84396/12**	**61/36**	**001/0**
	**NBW**	**53**	**1698/53**	**49548/7**	**39/70**	

**Table 3:** comparing developmental scores regarding sex

**sex**	**Developmental status**	**groups**	**NUM**	**MEAN**	**SD**	**Mean Rank**	**P**
**male**	**Communication**	**VLBW**	**22**	**59/51**	**17**	**41/21**	**048/0**
		**NBW**	**28**	**04/58**	**14/3**	**71/28**	
	**Gross motors**	**VLBW**	**22**	**45/50**	**97/16**	**82/18**	**001/0**
		**NBW**	**28**	**11/59**	**95/1**	**75/30**	
	**Fine motors**	**VLBW**	**22**	**23/40**	**42/18/**	**61/17**	**0001/0**
		**NBW**	**28**	**43/56**	**05/7**	**70/31**	
	**Socio-personal**	**VLBW**	**22**	**18/50**	**85/16**	**23/26**	**742/0**
		**NBW**	**28**	**75/53**	**02/5**	**93/24**	
	**Problem-solving**	**VLBW**	**22**	**27/37**	**86/14**	**23/15**	**0001/0**
		**NBW**	**28**	**14/53**	**14/7**	**57/33**	
**female**	**Communication**	**VLBW**	**31**	**84/53**	**82/8**	**37/24**	**021/0**
		**NBW**	**25**	**80/57**	**56/3**	**62/33**	
	**Gross motors**	**VLBW**	**31**	**52/16**	**60/13**	**47/24**	**017/0**
		**NBW**	**25**	**58**	**56/4**	**50/33**	
	**Fine motors**	**VLBW**	**31**	**87/39**	**04/14**	**97/20**	**0001/0**
		**NBW**	**25**	**32/54**	**62/10**	**84/37**	
	**Socio-personal**	**VLBW**	**31**	**06/53**	**54/10**	**87/25**	**151/0**
		**NBW**	**25**	**20/56**	**06/5**	**76/31**	
	**Problem-solving**	**VLBW**	**31**	**34/42**	**97/10**	**34/21**	**0001/0**
		**NBW**	**25**	**20/53**	**02/8**	**38/37**	

## Discussion

This is the first study conducted on VLBW and NBW children in Iran. The results showed the importance of childhood developmental follow-ups in VLBW children and mentioned VLBW as an important risk factor in five-year-old children. There was no significant difference between the groups regarding sex; therefore, it seems that sex played no important role in developmental delay. In VLBW children, the highest rate (32.1%) of developmental delay was found in fine motor skills, followed by problem-solving (18.9%) and gross motor (11.3%) skills, respectively. However, NBW children showed no significant developmental delay in communication, gross motor, and problem-solving skills. Overall, 3.80% and 1.9% of NBW children had fine motor and social/personal development delays, respectively. A higher rate of developmental delay in all domains of ASQ II, except the social/personal domain, was found in VLBW children. 

In this regard, a study by Karimi et al., which compared developmental delay in moderately-low-birth-weight (MLBW) children (1500-2499 g) and NBW children, showed the lower mean scores of all developmental domains in the MLBW group. They also mentioned LBW as a risk factor for developmental delay in five-year-old children (10). They observed significant delays in problem-solving (26%), gross motor (6%), and fine motor (9.3%) skills of MLBW children, which is consistent with our results and emphasize the effect of severe LBW on childhood developmental status. Comparison of the study by Karimi et al. with the present study revealed that the higher rate of delay in problem-solving skills might be a result of severe LBW, as well as the socio-environmental and family status of children (10).

In another study, the results showed that prematurity and history of VLBW were significantly related to motor disabilities in seven-year-old children (12). Also, a study by Reuner et al., which prospectively assessed 65 premature infants and 41 term infants up to adolescence, showed the higher rate of developmental delay in attending school among VLBW children and the lower rate of graduation (13). However, Datar et al., who compared the motor and mental development of VLBW and MLBW children with that of NBW children, showed that LBW had an insignificant negative effect on the child’s motor and mental development in the first two years of life (14).

Moreover, Boardmann et al. showed that birth weight had a significant relationship with the developmental status. The negative effect of birth weight was significantly higher in VLBW children as compared to MLBW children (15). Also, in a study by Schendel et al., which compared the developmental status of 15-month-old children, using the Denver Developmental Screening Test 2, the higher risk of moderate or severe developmental delay in VLBW children was reported (16). Besides, in a study by Zhang et al. assessing preterm infants, abnormal and severe neurological development problems were found in 29% and 12.4% of the patients, respectively (17). Further studies by Ballot et al. and Pietz et al. reported similar results (18, 19).

According to the present results, a higher risk of developmental delay was observed in VLBW infants as compared to NBW infants; therefore, it is recommended to perform developmental screening tests for timely detection of high-risk children and early diagnostic and therapeutic interventions. Further prospective cohort studies with a larger sample size over a longer follow-up period is recommended in VLBW children, using different screening tests. Also, assessing the etiology of developmental delay in VLBW children is highly suggested. Finally, researchers are recommended to conduct routine developmental assessments of VLBW in care programs for children younger than five years. 
